# The Impact and Mechanism of a Novel Allosteric AMPA Receptor Modulator LCX001 on Protection Against Respiratory Depression in Rodents

**DOI:** 10.3389/fphar.2019.00105

**Published:** 2019-02-19

**Authors:** Wei Dai, Xiang Gao, Dian Xiao, Yu-Lei Li, Xin-Bo Zhou, Zheng Yong, Rui-Bin Su

**Affiliations:** ^1^State Key Laboratory of Toxicology and Medical Countermeasures, Beijing Institute of Pharmacology and Toxicology, Beijing, China; ^2^Laboratory of Computer-Aided Drug Design and Discovery, Beijing Institute of Pharmacology and Toxicology, Beijing, China

**Keywords:** respiratory depression, glutamate, AMPA receptor modulator, ampakine, analgesics and sedative drugs

## Abstract

Analgesics and sedative hypnotics in clinical use often give rise to significant side effects, particularly respiratory depression. For emergency use, specific antagonists are currently administered to counteract respiratory depression. However, antagonists are often short-lasting and eliminate drug generated analgesia. To resolve this issue, novel positive AMPA modulators, LCX001, was tested to alleviate respiratory depression triggered by different drugs. The acetic acid writhing and hot-plate test were conducted to evaluate analgesic effect of LCX001. Binding assay, whole-cell recording, live cell imaging, and Ca^2+^ imaging were used to clarify mechanism and impact of LCX001 on respiratory protection. Results showed that LCX001 effectively rescued and prevented opioid (fentanyl and TH-030418), propofol, and pentobarbital-induced respiratory depression by strengthening respiratory frequency and minute ventilation. The acetic acid writhing test and hot-plate test revealed potent anti-nociceptive efficacy of LCX001, in contrast to other typical ampakines that did not affect analgesia. Furthermore, LCX001 potentiated [^3^H]AMPA and L-glutamate binding affinity to AMPA receptors, and facilitated glutamate-evoked inward currents in HEK293 cells stably expressing GluA2(R). LCX001 had a typical positive modulatory impact on AMPAR-mediated function. Importantly, application of LCX001 generated a significant increase in GluA2(R) surface expression, and restrained opioid-induced abnormal intracellular Ca^2+^ load, which might participate in breathing modulation. Our study improves therapeutic interventions for the treatment of drug induced respiratory depression, and increases understanding of potential mechanism of AMPA receptor modulators.

## Introduction

Analgesics and sedative drugs are irreplaceable for clinical treatment, and administered for the induction and maintenance of anesthesia (Searle and Sahab, 1993; Kimura and Haji, 2014). However, different side effects arise from body intolerance, drugs overdoses and combination therapy (Jungquist et al., 2011). Analgesic and sedative drug-induced respiratory depression is still an unresolved and significant problem in clinical treatment and it can be a leading cause of death (Dahan et al., 2010). The opioids directly suppress respiratory activity by activating the μ-opioid receptor in the brainstem respiratory generating and regulating center (Greer et al., 1995; Gray et al., 1999). Propofol and pentobarbital cause acute respiratory depression, which is partially attributed to stimulation of γ-aminobutyric acid (GABA) receptors and an inhibitory effect on the rhythmic respiratory drive in brainstem (Trapani et al., 2000; Sigel, 2002). Due to the non-specificity of receptor activation, respiratory depression is commonly experienced with analgesic and sedative drug use (Dai et al., 2017). In addition, abuse of opioids is a developing public health and social issue. Opioids are frequently overcommitted and used in combination with other sedative substances (alcohol, zolpidem, etc.) by abusers. These scenarios enormously increase the risk of death triggered by respiratory depression (Hasegawa et al., 2014; Fala and Welz, 2015). Antagonists (naloxone, flumazenil etc) are effectively administered to rescue varying degrees of suppressed respiration. However, limitations of special antagonists still exist. For instance, injection of naloxone often eliminates the opioids analgesia (Olofsen et al., 2010). Some severe respiratory depression cannot be overcome by naloxone, and artificial ventilation is deem to be more beneficial (Van Dorp et al., 2007; Roozekrans et al., 2015). Flumazenil has good safety in the clinical application, whereas the continuous administration of intravenous drip is required on account of its short half-life. Arrhythmia and convulsion can be produced by flumazenil at high doses (Roncari et al., 1986; Amrein et al., 1987).

New pharmacological approaches to alleviate analgesic and sedative drug-induced depression of respiration have been sought. Special allosteric amino-3-hydroxy-5-methylisooxazole-4-propionic acid (AMPA) receptor modulators, belonging to the ampakine family, positively promote the function of AMPA receptors by strengthening the duration of the glutamate-induced inward current and slowing down the deactivation or desensitization rates of the AMPA ion channel (Harms et al., 2013; Weeks et al., 2014). Ampakines, therefore, promote drive to central respiratory motor neurons and can reverse severe respiratory depression or life-threatening apnea. Typical AMPA modulator CX546 effectively enhances the central respiratory drive and output in medullary slices, and counters opioid-induced respiration suppression (Ren et al., 2006). *In vivo*, CX717 remarkably alleviates severe respiratory depression induced by fentanyl or the combination of alcohol and barbiturates in the plethysmography tests (Ren et al., 2009; Ren et al., 2015). The trials in healthy men reveal that CX717 has a noteworthy treatment effect on alfentanil-induced respiratory depression without interfering with the opioid analgesia (Oertel et al., 2010). CX717 has been through several Phase I and II clinical trials without signs of severe side-effects (Lorier et al., 2010; ElMallah et al., 2015). Synthesized allosteric AMPA modulators are considered promising for alleviation of respiratory depression.

**FIGURE 1:**
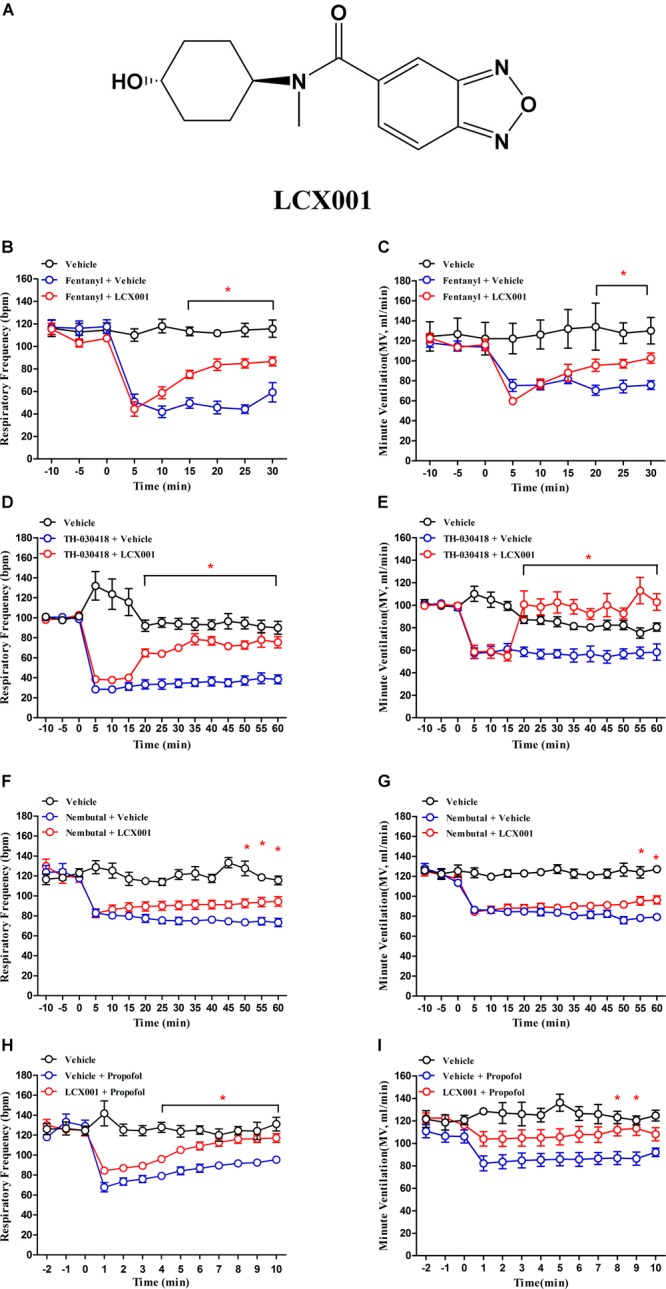
Administration of LCX001 (10 mg/kg, i.v.) protected against opioid, propofol and pentobarbital (Nembutal) induced respiratory depression in unanaesthetised adult rats. The data, acquired from whole body plethysmography, showed the time-varying changes of respiratory frequency (bpm) and minute ventilation (MV) in response to opioid, propofol and pentobarbital administration after post- or pre-treatment of vehicle (20% HPCD) or LCX001. **(A)** Chemical structure of the modulator, LCX001 (N-((1r,4r)-4-hydroxycyclohexyl)-N-methylbenzo[c][1,2,5]oxadiazole-5-carboxamid. **(B,C)** 5 min after fentanyl (120 μg/kg, s.c.), injection of LCX001 (10 mg/kg, i.v.) effectively countered the inhibition of respiratory frequency and minute ventilation. **(D,E)** LCX001 (10 mg/kg, i.v.) administered 5 min after TH-030418 (20 μg/kg, i.v.) alleviated the reduction of respiratory parameters. **(F,G)** 5 min after Nembutal (80 mg/kg, i.p.) administration, LCX001 (10 mg/kg, i.v.) ameliorated respiratory depression by increasing respiratory rate and minute ventilation. **(H,I)** LCX001 (10 mg/kg, i.v.) administered 10 min before propofol (30 mg/kg, i.v.) reversed the acute reduction of respiratory frequency and minute ventilation. ^∗^Significant difference from fentanyl, TH-030418, Nembutal and propofol groups by two-way ANOVA, Bonferroni *post-hoc* tests; LCX001 (^∗^*P* < 0.05) relative to the fentanyl, TH-030418, Nembutal and propofol groups; mean ± SEM, each symbol represented data from six male SD rats.

In this study, we characterize an active ampakine compound LCX001 (Figure 1A) from the RespireRx (Cortex) patents. Application of LCX001 promotes a 21% increase in the amplitude of synaptic currents in the rat dentate gyrus (Street et al., 2008; Mueller and Street, 2012). No further research on LCX001 has been reported. We conjectured that LCX001 might play a role in the protection against respiratory depression. In accordance with this hypothesis, plethysmography measurements were taken to detect the effect of LCX001 on respiratory depression caused by several analgesics and sedative hypnotics. Moreover, the possible anti-nociceptive impact of LCX001 was evaluated by the acetic acid writhing and hot-plate tests. To explore the mechanisms of respiratory failure rescue, binding assays and whole-cell recordings were used to investigate the pharmacological quality of LCX001. Specifically, live cell imaging and Ca^2+^ imaging were taken to detect the impact of LCX001 on AMPA receptors trafficking and intracellular calcium ion concentration in neurons. We believed that LCX001 might promote ampakines to be a therapeutic option for protection against respiratory depression.

## Materials and Methods

### Animals

Sprague-Dawley (SD) rats (male, 220–250 g) and Kunming (KM) mice (male or female, 20–22 g) were purchased from the Beijing Animal Centre (Beijing, China). All animals were housed with food and water *ad libitum*. The room was maintained at 25 ± 1°C with an alternating 12 h circadian cycle. All procedures were conducted in accordance with the Animal Care and Use Committee of the Beijing Institute of Pharmacology & Toxicology (Beijing, China). The study protocols were approved according to guidelines on the ethical use of animals set by the National Institutes of Health (Bethesda, MD, United States).

### Pharmacological Agents

LCX001 was provided by the Laboratory of Computer-Aided Drug Design & Discovery, Beijing Institute of Pharmacology and Toxicology (Xiao et al., 2016). Fentanyl (Sigma, Beijing, China) and TH-030418 were administered to generate opioid-induced respiratory depression models. TH-030418 (N-methyl-7a-[(R)-1-hydroxy-1-methyl-3-(thien-3-yl)-propyl]-6,14-endo-ethanotetrahydronororipavine) was a thienorphine derivative, synthesized by our institute. Compared with morphine or dihydroetorphine, TH-030418 displayed non-selective and high binding affinity to all subtypes of opioid receptors (Wen et al., 2011). TH-030418 also exerted a strong and long-acting analgesic effect. Ampakines, LCX001 and CX614 (Tocris, Bristol, United Kingdom), were dissolved in a 20% hydroxypropyl-β-cyclodextrin (HPCD; Sigma-Aldrich, Beijing, China) 0.45% saline solution. Propofol was purchased from AstraZeneca (Viale Dell’industria, Italy). Pentobarbital (Nembutal; Merck, Beijing, China), fentanyl, TH-030418, and morphine hydrochloride (Qinghai Pharmaceutical Factory, Xining, China) were dissolved in 0.45% saline solution at room temperature and used at different doses. The volume of each dose was 1 ml/kg for SD rats and 10 ml/kg for KM mice.

### Plethysmography Recording

Whole-body plethysmographs (EMKA Technologies, Paris, France) of breathing frequency and depth were performed on unrestrained rodents to evaluate the protection effect of LCX001 against respiratory depression induced by fentanyl, TH-030418, propofol and pentobarbital (Nembutal). Adult male SD rats were placed in the transparent plexiglass instrument consisting of inflow and outflow ports for the continuous delivery of fresh air and removal of expired carbon dioxide. Baseline was produced approximately 30 min after acclimatization of rats to the apparatus environment. Pressure changes were measured with a pressure transducer and a signal conditioner. Each group contained six male SD rats. The control groups incorporated (1) vehicle (20% HPCD, intravenous, i.v.) at 5 min after fentanyl (120 μg/kg, subcutaneous, s.c.), TH-030418 (20 μg/kg, i.v.) or pentobarbital (Nembutal, 80 mg/kg, intraperitoneal, i.p.) separately; (2) vehicle (20% HPCD, i.v.) at 10 min before propofol (30 mg/kg, i.v.); (3) vehicle alone. The experimental groups were post-administered with LCX001 (10 mg/kg, i.v.) at 5 min after fentanyl (120 μg/kg, s.c.), TH-030418 (20 μg/kg, i.v.), or pentobarbital (Nembutal, 80 mg/kg, i.p.), and pre-treatment of LCX001 were conducted at 10 min before propofol (30 mg/kg, i.v.) because of the short-lasting propofol-induced respiratory depression (15–25 min). Respiration data were acquired and analyzed using IOX software (EMKA Technologies, Paris, France).

### Acetic Acid Writhing Test and Hot-Plate Test

The analgesic effect of LCX001 was evaluated in the acetic acid-induced writhing test. Male KM mice were injected with acetic acid (0.6%, 0.4 ml/20 g i.p.) and then placed in a transparent plastic cage for observation. The number of writhes was counted and recorded 5 min after acetic acid administration for 15 min. The writhe movement was defined as contraction of the abdominal muscles causing hind limb stretching. Male KM mice (*n* = 70) were randomly and equally divided into seven groups. In the control groups, morphine (2.5 mg/kg, s.c.) or vehicle (20% HPCD, i.v.) was administered 30 min prior to injection of 0.6% acetic acid. The pre-treatment of LCX001 (12 mg/kg, iv) at 10 min before morphine (2.5 mg/kg, s.c.) was also set as a control group, which was followed by injection of 0.6% acetic acid after 30 min. In the experimental groups, LCX001 (1.5, 3, 6, or 12 mg/kg, i.v.) were administered 10 min before 0.6% acetic acid. Analgesia percentage was expressed using the following formula: Inhibition ratio (%) = 100 × (number of control-number of experiment)/number of control.

The hot plate test was performed to determine the anti-nociceptive effect of LCX001. The temperature of the hot plate (Hugo Sachs Elektronik-Harvard Apparatus GmbH, March-Hugstetten, Germany) was set and maintained at 55 ± 0.5°C. The latency of hind paw licking was evaluated and recorded as the response to nociception. Baseline response latencies of under 5 s or over 30 s were eliminated from the test. Female KM mice (*n* = 70) were randomly divided into seven groups of 10 each. The hot-plate response latency was measured at 30 min after morphine (10 mg/kg, s.c.) injection, and at 10 min after LCX001 (1.5, 3, 6, or 12 mg/kg, i.v.) or vehicle (20% HPCD, i.v.) administration. The pre-treatment of LCX001 (12 mg/kg, iv) at 10 min before morphine (10 mg/kg, s.c.) was also set as a control group to investigate the impact of LCX001 on anti-nociceptive effect of morphine. Anti-nociceptive data were quantified according to Maximum Possible Effect (MPE), and calculated as: MPE (%) = [(T1–T0)/(T2–T0)] × 100 (T0 and T1 represent latency times of hind-paw licking before and after treatment. T2 was the cut-off time, which was set at 60 s to prevent injury to the animal’s paw).

### [^3^H]AMPA Binding

Adult male SD rats (220–250 g) were euthanized by decapitation. The brainstems were dissected and homogenized for 2 min in 15 volumes of ice-cold 50 mM Tris–HCl buffer (pH 7.4) at 4°C. The homogenates were centrifuged at 1,000 g for 10 min, and the supernatants were collected and centrifuged at 40,000 g for 20 min. The pellets were dispersed in 50 mM Tris-HCl pH 7.4 and centrifuged at 40,000 g for 20 min. The last procedures were repeated a third time with a total of four washes to eliminate endogenous glutamate from the tissue. The resulting pellets were suspended in eight volumes of 0.3 M sucrose, then stored at –70°C until use.

Tissue membranes were washed twice in 50 mM Tris-HCl pH 7.4, and the resultant membranes resuspended in 50 mM Tris-HCl pH 7.4 in the presence of 100 mM KSCN. The dispersed tissue membranes were incubated with 200 nM [^3^H]AMPA (58 Ci/mmol, PerkinElmer, Shanghai, China) and LCX001 at different doses (10^-9^–10^-4^ M) in a final volume of 1 ml. The binding reaction was performed in disposable glass culture tubes at 25°C for 60 min. Bound [^3^H]AMPA was separated from the free ligand by filtration under reduced pressure with GF/C filters presoaked in 0.2% polyethyleneimine. The pellets were washed with ice-cold Tris-HCl pH 7.4 three times and dried with a piece of tissue paper. Nonspecific binding was determined by 1 mM nonradioactive glutamate. The binding activity on the filters was detected using a LS6500 scintillation counter (Beckman, Shanghai, China). For saturation binding with [^3^H]AMPA, LCX001 (10^-4^ M) was added in the resuspended assay with changing concentrations of [^3^H]AMPA from 1 to 400 nM.

### Plasmid Construction, Culture, and Stable Transfection of HEK293 Cell

A wild-type rat AMPA-GluA2(R)_flop_ cDNA corresponding to the entire coding region was generated and cloned into the pcDNA^TM^3.1(+) vector (Invitrogen, China, Catalog no. V790-20) by Sangon Biotech Co., Ltd. (Shanghai, China). Human embryonic kidney 293 (HEK293) cells (purchased from the National Infrastructure of Cell Line Resource of China) were cultured in DMEM (Gibco, Shanghai, China) supplemented with 10% fetal calf serum (Hyclone, Beijing, China) and grown in an incubator at 37°C, in a 5% CO_2_, 70% humidified atmosphere. For stable transfection, HEK293 cells (3 × 10^4^ cells/well) were seeded in 24-well plates the day before transfection. The cells were grown to 70–90% confluency, and the transfection was performed by adding 1 μg of GluA2 expressing vector per well. Twenty-four hours after transfection, the normal medium was replaced with DMEM/F12 (Gibco, Shanghai, China) growth medium supplemented with 1.0 mg/ml G418 (Gibco, Shanghai, China) and cultured for 7 days. The surviving cells were pelleted and dispersed (100 cells/20 ml), and then seeded into 96-well plates in DMEM/F12 medium containing 500 μg/ml G418. Discrete colonies were picked after 15 days of selection. Western blotting and whole-cell patch-clamp recording were performed to test expression of the AMPA receptor GluA2 subunit and formation of an AMPA-GluA2 ion channel. G418 was kept at 500 μg/ml for the maintenance of stably transfected clones.

### Whole-Cell Patch-Clamp Recording

HEK293 cells that stably expressed AMPA-GluA2 receptors were seeded into the recording chamber 2 days before patch-clamp recordings, and the experiments were conducted in whole-cell configuration. Thin-walled borosilicate glass (Sutter Instrument, CA, United States) was pulled to make patch pipettes. The patch pipettes had open tips with resistance of 4–8 MΩ and were filled with internal electrode solution (mM): 135 CsCl (Sigma, Beijing, China), 10 CsF (Tocris, Bristol, United Kingdom), 10 HEPES (Amresco, United States), 5 Cs4BAPTA (Tocris, Bristol, United Kingdom), 1 MgCl_2_, 0.5 CaCl_2_, pH 7.2. The external bath solution consisted of (mM): 135 NaCl, 5 KCl, 10 HEPES, 1 MgCl_2_, 1.8 CaCl_2_, pH 7.35. The holding potential was set as -80 mV by an Axopatch-700B amplifier (Axon Instruments, CA, United States), and pClamp8 software (Axon Instruments, CA, United States) was used for data acquisition. Currents were recorded and digitized at 10 kHz. A fast-solution switch system was utilized for agonist (L-glutamate, Novabiochem, Darmstadt, Germany) application at varying concentrations, which allowed constant exchange of bath solutions to the cells, and stopped momentarily on the application of agonist or drugs. LCX001 and CX614 were dissolved in dimethyl sulfoxide (DMSO) before dilution with external bath solution (DMSO final concentration 0.5%). Dose-response curves were fitted to the Hill equation using Origin 9.0 software.

### Primary Medulla Oblongata Neuronal Cultures

Isolated brainstems were prepared from neonatal (P0-P1) SD rats using previously described methods (Mazza et al., 2000). A microscope was used for tissue dissection under aseptic conditions. The rat pups were decapitated, and the brainstems were rapidly removed. After microdissection of the cerebellum and pons from the brainstem, the medulla oblongata was separated and transferred to a new chamber. The tissue containing the medulla oblongata was minced and digested enzymatically with 0.25% trypsin for 15 min. The medulla oblongata cells were filtered, then dispersed and seeded in DMEM medium supplemented with 10% fetal calf serum and 10% equine serum (ExCell Bio, Shanghai, China). Twenty-four hours after seeding, the medium was replaced by Neurobasal (Gibco, Shanghai, China) Medium with B27 (Gibco, Shanghai, China) supplement (50:1). The medulla oblongata cells were fed on days 3–7 with a half volume change of Neurobasal/B27 medium containing Ara-C (3–5 μM) to inhibit glial growth. The cells were cultured for 10–15 days prior to use.

### Live Cell Imaging

The GluA1 or GluA2 AMPA receptor subunits tagged with super ecliptic pHluorin (SEP) were used to investigate the trafficking and accumulation of AMPA receptors on the surface of neurons in real-time. Plasmids containing SEP-GluA1 and SEP-GluA2(R) were purchased from Addgene (plasmid #24000, plasmid #24001). Recombinant adenoviruses carrying SEP-GluA1 or SEP-GluA2(R) were constructed by Hanbio biotechnology (Shanghai, China) for neuronal transfection and SEP expression, and the plaque-forming units (PFU) were 1.26 × 10^10^/ml. Dissociated primary medulla oblongata neurons were prepared as described above and seeded and cultured using 20 mm Poly-D-lysine-coated dishes with inner glass coverslips (NEST, Jiangsu, China) for the preparation of live cell imaging. The primary neurons, cultured for 10 days, were transfected using recombinant adenovirus with incubation at 37°C, in a 5% CO_2_, 70% humidified atmosphere for 6 h. The medium containing virus was replaced with normal medium, and imaging was performed 96 h after transfection. Artificial cerebrospinal fluid (ACSF) was prepared as bathing solution containing (mM): 120 NaCl, 5 KCl, 2 CaCl_2_, 1.2 MgCl_2_, 30 D-glucose, 25 HEPES, 1 tetrodotoxin, pH 7.4. For live neuron imaging, transfected cells were identified by GFP fluorescence in the bathing ACSF solution. Images were taken using the 20 × objective lens (Hamamatsu C9100-50) of an inverted confocal laser scanning microscopy system (PerkinElmer, UltraVIEW VoX, Shanghai, China) at 37°C. SEP fluorescence was achieved from a 488 nm laser line. The time-lapse imaging of SEP-GluA1 and SEP-GluA2 was set with 1s intervals and 100 ms exposure times for 10 min. For testing local stimulation, LCX001 or CX614 (47 μM final) was added to the standard solution 2 min after the commencement of imaging. Regions of interest (ROIs) were chosen from areas of diffuse fluorescence in the shaft and spines for comparison before and after drug treatment.

### Ca^2+^ Imaging

Dissociated primary medulla oblongata neurons were prepared as described above. The primary neurons were seeded using 20 mm Poly-D-lysine-coated dishes with inner glass coverslips (NEST, Jiangsu, China) and cultured for 15 days. On the day of the Ca^2+^ imaging experiment, Fluo-4 NW dye buffer was prepared by adding 10 ml Hank’s Balanced Salt Solution (HBSS) containing 20 mM HEPES, 2.5 mM probenecid, and 1.5 mM CaCl_2_ to one bottle of Fluo-4 NW dye mix (Fluo-4 NW Calcium Assay Kits, Thermo fisher, F36206, Shanghai, China). The normal medium for neurons was replaced with Fluo-4 NW dye buffer and incubated in a 37°C atmosphere for 45 min. The assay was initiated when a baseline was produced, and followed by the addition of L-glutamate (18 μM final) at 1 min. Three minutes after the commencement of imaging, fentanyl or TH-030418 (42 μM final) was added, and the neurons were treated with LCX001 or CX614 (40 μM final) 7 min after the commencement of imaging. To evaluate the possible effect of modulators, changes in intracellular Ca^2+^ were recorded using the 20 × objective lens (Hamamatsu C9100-50) of an inverted confocal laser scanning microscopy system (PerkinElmer, UltraVIEW VoX, Shanghai, China) at 37°C. Fluorescence of Ca^2+^ imaging was obtained from a 488 nm laser line. Time-lapse imaging was set with 1 s intervals and 100 ms exposure times for 10 min.

### Statistical Analysis

Data are presented as the mean ± standard error of the mean (SEM). In the acetic acid writhing and hot-plate tests, experiments were performed blind. Compounds were injected by one researcher and the behavior or data was recorded by another researcher. The significance of change in respiratory parameters was evaluated by repeated measures two-way ANOVA (dose × time) followed by Bonferroni *post-hoc* tests for comparisons between groups. In the analysis of the acetic acid writhing test, hot-plate test and [^3^H]AMPA binding assay, statistical evaluation between the treated groups and control group was performed by one-way ANOVA followed by Dunnett’s test. For saturation binding with [^3^H]AMPA, *K_d_* and *B_max_* values of different groups were obtained from fits to saturation binding curves with the KaleidaGraph programme. For whole-cell patch-clamp recordings, dose-response curves were fitted to the Hill equation using Origin 9.0 software (Originlab, MA, United States) and statistical significance was determined by one-way ANOVA followed by Dunnett’s test. For live cell imaging, two-way ANOVA followed by Bonferroni *post-hoc* tests was used to compare the treated groups and vehicle group. A *P* value of less than 0.05 was considered to represent a statistically significant difference.

## Results

### LCX001 Alleviates Fentanyl, TH-030418, Propofol, and Pentobarbital-Induced Respiratory Depression in Rats

Whole body plethysmography measurements are used to detect spontaneous respiratory parameters generated by unanaesthetised male SD rats. As shown in Figure 1, administration of fentanyl, TH-030418, propofol and pentobarbital (Nembutal) markedly suppressed respiration. Nembutal (80 mg/kg, i.p.) and propofol (30 mg/kg, i.v.) triggered a reduction in respiratory frequency (70.3 ± 2.7% of control, 53.6 ± 5.8% of control, Figure 1F,H). The μ-opioid receptor agonist fentanyl (120 μg/kg, s.c.) and TH-030418 (20 μg/kg, i.v.) produced a more severe suppressive effect on respiratory rate (43.4 ± 5.8% of control, 28.9 ± 3.3% of control, Figure 1B,D), and the suppressed respiration lasted for more than 30 min (Figure 1B,D). 5 min after opioid and pentobarbital treatment, post-administration of LCX001 (10 mg/kg, i.v.) significantly alleviate respiratory depression by increasing the respiratory frequency and minute ventilation (Figure 1B–G, ^∗^*P* < 0.05). In consideration of the short-lasting propofol-mediated depression (within 15–25 min), LCX001 (10 mg/kg, i.v.) was injected 10 min before propofol administration. The pre-treatment of LCX001 also markedly supported pulmonary function after propofol-induced depression (Figure 1H,I, ^∗^*P* < 0.05).

### Anti-nociceptive Effects of LCX001

**FIGURE 2:**
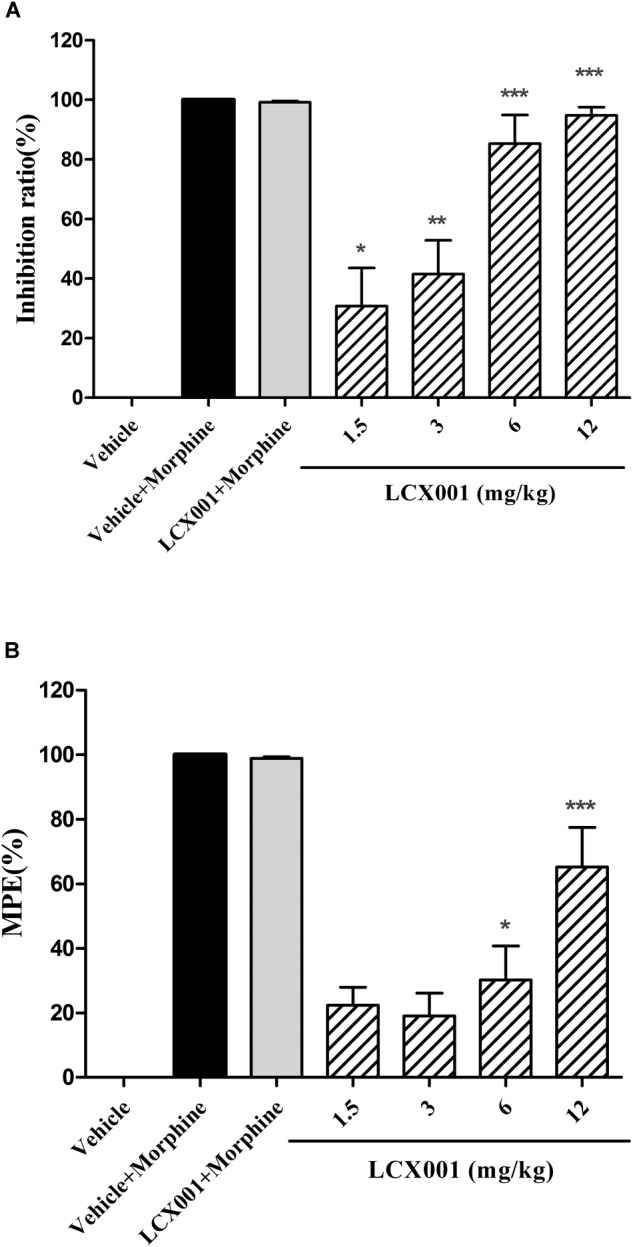
Anti-nociceptive effect of LCX001. Morphine (2.5 or 10 mg/kg, s.c.) was used as positive control drug. **(A)** Effect of LCX001 in the writhing test. 10 min before 0.6% acetic acid treatment, LCX001 administration (1.5, 3, 6, and 12 mg/kg, i.v.) significantly inhibited acetic acid-induced writhing compared to vehicle (20% HPCD, i.v.); mean ± SEM, each group contained 10 male KM mice. **(B)** Effect of LCX001 in the hot-plate test. LCX001 at 6 and 12 mg/kg (i.v.) markedly increased %MPE after the hot-plate stimulation as compared with the vehicle control group. ^∗^Significant difference by one-way ANOVA, Dunnett’s test (LCX001 treatment groups vs. vehicle control group;^∗^*P* < 0.05, ^∗∗^*P* < 0.01 and ^∗∗∗^*P* < 0.001); mean ± SEM, each group contained 10 female KM mice.

From the Acetic acid writhing testing, as shown in Figure 2A, a 2.5 mg/kg dose of morphine (s.c.) significantly reduced the number of abdominal constrictions of male KM mice compared with the vehicle (20% HPCD, i.v.) group, and the inhibition rate reached entirely 100%. LCX001 in doses of 1.5, 3, 6, and 12 mg/kg inhibited acetic acid-induced writhing by 30.76 ± 12.75%, 41.43 ± 11.32%, 85.35 ± 9.53%, and 94.78 ± 2.74%, respectively. These results indicated that LCX001 had a significant anti-nociceptive effect at 1.5 mg/kg or higher doses (Figure 2A, ^∗^*P* < 0.05), and a 10 mg/kg dose of LCX001, similar to morphine at 2.5 mg/kg, produced a marked analgesic effect on writhing behaviors induced by acetic acid.

In the hot plate test, Morphine (10 mg/kg, s.c.) markedly reduced hind paw licking time of female KM mice and had an increased %MPE of about 100% after hot-plate stimulation (Figure 2B). Administration of LCX001 (i.v.) at 1.5 and 3 mg/kg exerted no effect on the %MPE compared with the vehicle (20% HPCD, i.v.) treated group (Figure 2B, *P* > 0.05). However, intravenous injection of LCX001 at 6 and 12 mg/kg increased the level of %MPE by 30.18 ± 10.56% and 65.27 ± 12.22%, respectively (Figure 2B, ^∗^*P* < 0.05). The treatment of LCX001 (12 mg/kg) exerted no impact on the analgesic effect of morphine (Figure 2A,B, vehicle+morphine vs. LCX001+morphine, *P* > 0.05). These results demonstrated that LCX001 at doses of 6 mg/kg or higher produced anti-nociceptive behavior in both acetic acid writhing and hot-plate tests.

### LCX001 Potentiates [^3^H]AMPA Binding

**FIGURE 3:**
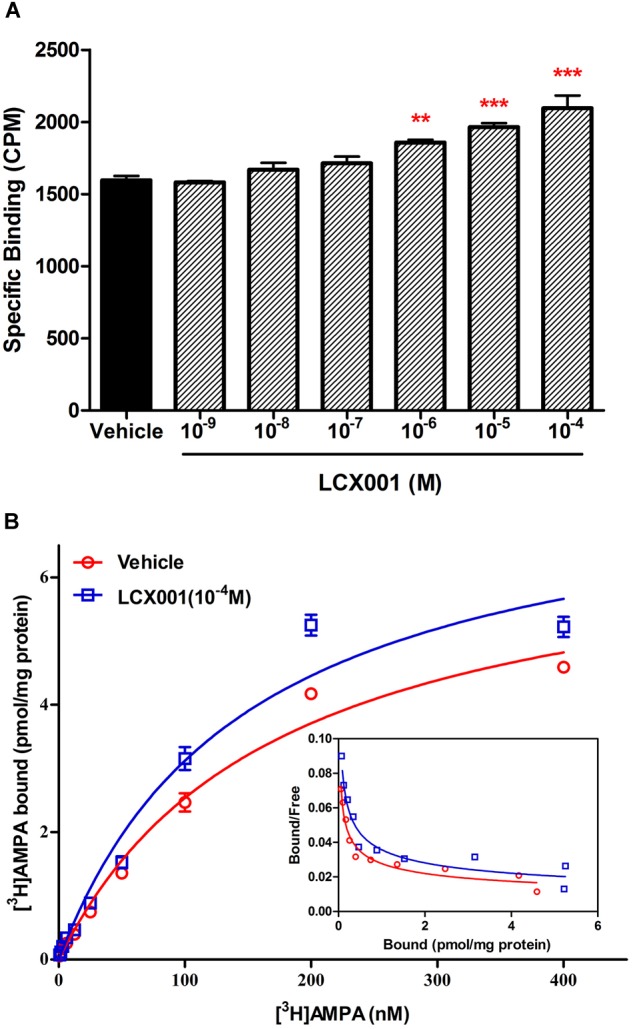
LCX001 facilitated [^3^H]AMPA binding. **(A)** Effects of preincubation of LCX001 at increasing doses (10^-9^–10^-4^ M) on [^3^H]AMPA binding. The specific binding of [^3^H]AMPA to AMPA receptors in tissues was significantly improved in the presence of LCX001 at 10^-6^, 10^-5^, and 10^-4^ M. ^∗^Significant difference by one-way ANOVA, Dunnett’s test (LCX001 treatment groups vs. vehicle group;^∗∗^*P* < 0.01, ^∗∗∗^*P* < 0.001); mean ± SEM, three separate experiments were performed in triplicate. **(B)** Saturation binding curves and Scatchard plots (inset) of [^3^H]AMPA binding in the preincubation and absence from LCX001 (10^-4^ M). The concentrations of [^3^H]AMPA were set to 400 nM. The results were analyzed by one-site logistic curve-fitting equation, and the *K_d_* and *B_max_* values given in the text indicated that binding affinity and the number of binding sites were increased after treatment of LCX001. The *K_d_* and *B_max_* values were individually mentioned in the text. Values are the mean ± SEM of results from six separate experiments.

In the [^3^H]AMPA binding assay, specific [^3^H]AMPA binding to AMPA receptors of tissue was measured as 1595 ± 30 (cpm/mg protein). Compared with the control group, specific [^3^H]AMPA binding was unchanged by pre-incubation with LCX001 at 10^-9^–10^-7^ M. However, pre-treatment of LCX001 at 10^-6^, 10^-5^, and 10^-4^ M significantly enhanced [^3^H]AMPA binding affinity to tissue membranes by 1857 ± 20, 1964 ± 27 and 2095 ± 87 (cpm/mg protein) respectively (LCX001 vs. vehicle control group, ^∗∗^*P* < 0.01 and ^∗∗∗^*P* < 0.001, Figure 3A). These results indicate that LCX001 at 10^-6^ M or higher doses increased the binding affinity of [^3^H]AMPA for AMPA receptors, and a 10^-4^ M dose of LCX001 was chosen to generate saturation curves of [^3^H]AMPA binding. Several [^3^H]AMPA concentrations up to 400 nM were performed in the saturation binding experiments to investigate the impact of LCX001 pre-incubation (10^-4^ M) on *K_d_* and *B_max_* parameters. The [^3^H]AMPA saturation binding results were analyzed using the direct-fit logistic equation, and the curve was best fitted as a one-site model. The *K_d_* and *B_max_* values were 171 ± 5 nM and 6.89 ± 0.11 pmol/mg of protein (Figure 3B). After pre-incubation with LCX001 at 10^-4^ M, the *K_d_* and *B_max_* values from direct fitting were 149 ± 7 nM and 7.77 ± 0.17 pmol/mg of protein (Figure 3B). From the analysis of *K_d_* and *B_max_* value in saturation curves, the binding affinity and the number of binding sites were significantly improved with LCX001 treatment (vehicle group vs. LCX001, student’s paired *t*-test, ^∗^*P* < 0.05).

### LCX001 Promotes Apparent Affinity of Glutamate and Increases Glutamate-Evoked Inward Currents in HEK293 Cells Stably Expressing GluA2(R)_flop_

**FIGURE 4:**
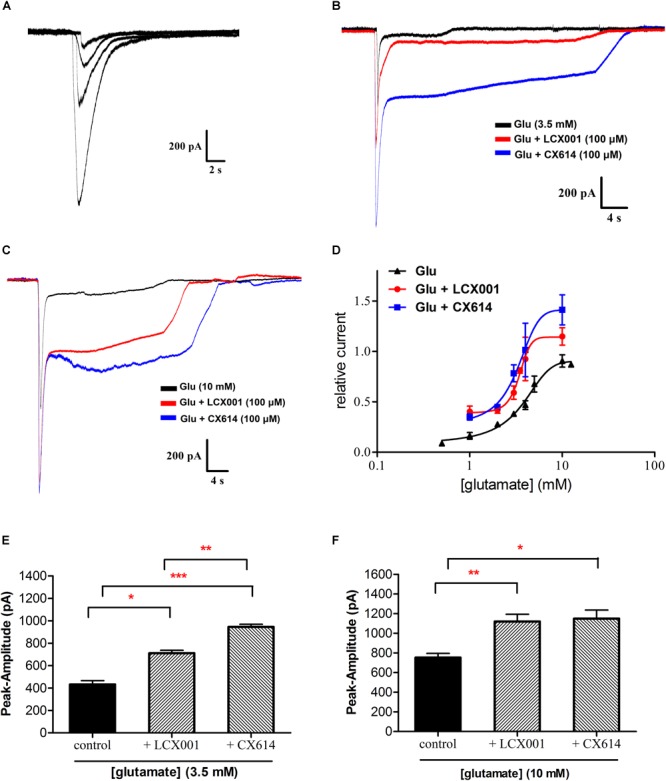
LCX001 facilitated apparent affinity of glutamate and increased glutamate-induced amplitudes. Representative traces were obtained from HEK293 cells expressing recombinant homomeric GluA2(R)_flop_ receptor. **(A)** Typical response to application of glutamate at increasing doses (1–5 mM) in a whole-cell clamp-patch recording. **(B)** 3.5 mM glutamate evoked currents in the absence and presence of 100 μM LCX001 or CX614. **(C)** 10 mM glutamate evoked currents in the absence and presence of 100 μM LCX001 or CX614. **(D)** Concentration-response profile of glutamate-mediated currents alone and in the presence of 100 μM LCX001 or CX614. Values of amplitudes were normalized to the current from 10 mM glutamate in the same patch. The data were fitted by a Hill-type equation, and the EC_50_ obtained from the fits was given in the text. The n_H_ values of glutamate alone and in the presence of LCX001 or CX614 were 0.3, 0.84, and 0.43 respectively. **(E,F)** 100 μM LCX001 and CX614 were efficacious at enhancing glutamate (3.5 and 10 mM) evoked peak amplitudes. ^∗^Significant difference by one-way ANOVA, Dunnett’s test (LCX001 or CX614 treatment group vs. control group;^∗^*P* < 0.05,^∗∗^*P* < 0.01 and ^∗∗∗^*P* < 0.001); mean ± SEM, *n* = 6.

Whole-cell voltage-clamp electrophysiology is conducted to assess the impact of LCX001 on HEK293 cells transfected with the cDNAs encoding recombinant homomeric GluA2 receptors. As a potent AMPA modulator, CX614 serves as a positive control. A concentration–response curve of glutamate evoked currents was obtained with increasing concentrations of glutamate (Figure 4A), and an effective concentration 50% of maximum response (EC_50_) value fitted with a Hill-type equation was 3.43 mM (Figure 4D). When cells were recorded in the presence of modulators, co-application of LCX001 caused a significant leftward shift in the dose-response curve for glutamate and the EC_50_ value of LCX001 treatment was 2.76 mM (Figure 4D). Consistent with the effect of LCX001, CX614 triggered leftward shifts in the curve and EC_50_ values of 1.69 mM (Figure 4D). However, LCX001 alone induced no current in the absence of glutamate. These results indicated that both LCX001 and CX614 altered and promoted agonists’ affinities of glutamate to homomeric GluA2 receptors. From the concentration–response curve of glutamate evoked currents, the doses producing a half maximal response (3.5 mM) and a maximal response (10 mM) were used to evaluate the potentiation of LCX001 on glutamate-evoked amplitudes. As shown in Figure 4B,C,E,F, LCX001 (100 μM) potentiated the glutamate (3.5 mM) response by 711 ± 20 (pA) as compared with that of 432 ± 27 (pA) in the control group (Figure 4B,E, ^∗^*P* < 0.05). However, 100 μM CX614 was more efficacious with a potentiation of 946 ± 16 (pA) on the glutamate (3.5 mM) response (Figure 4B,E, ^∗^*P* < 0.05). At 10 mM glutamate evoked amplitudes, LCX001 and CX614 at 100 μM increased the potency of glutamate induced currents by 1120 ± 60 and 1149 ± 71 (pA), respectively, compared with that of 752 ± 35 (pA) in the control group (Figure 4C,F, ^∗^*P* < 0.05). These data revealed that LCX001 markedly enhanced the potency of glutamate at 3.5 and 10 mM, and acted as a positive novel allosteric modulator.

### LCX001 Mediates Stabilization of Surface GluA2-Containing AMPARs

**FIGURE 5:**
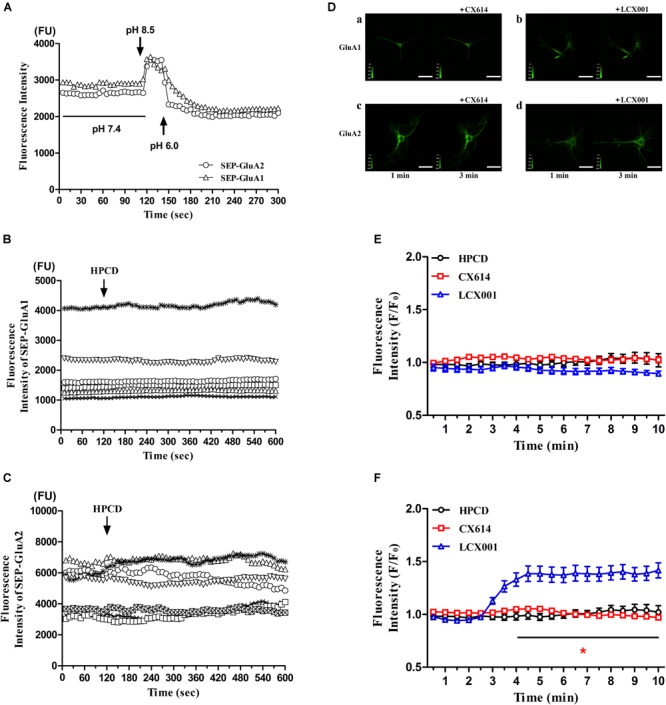
LCX001 increased surface expression of GluA2-containing AMPARs. **(A)** Time courses of SEP-GluA1 and SEP-GluA2 fluorescence in primary medulla oblongata neurons after exposure to extracellular alkali or acidic solutions. **(B,C)** SEP-GluA1 or SEP-GluA2 fluorescence time course after vehicle (20% HPCD) treatment at the time point of 2 min. The application of vehicle control had no influence on fluorescence intensity, *n* = 6. **(D)** Representative live cell images from primary medulla oblongata neurons transfected with SEP-GluA1 or SEP-GluA2 in the presence of LCX001 or CX614 (final dose of 47 μM). a. SEP-GluA1 treated with CX614 at 2 min, b. SEP-GluA1 treated with LCX001 at 2 min, c. SEP-GluA2 treated with CX614 at 2 min, d. SEP-GluA2 treated with LCX001 at 2 min. **(E,F)** Relative fluorescence intensity to the vehicle of pHluorin-GluA2 and GluA1 in response to LCX001 or CX614 treatment. Application of LCX001 at a final dose of 47 μM resulted in a significant increase in SEP-GluA2 fluorescence and enhanced stabilization of surface GluA2-containing AMPARs. ^∗^*P* < 0.05, two-way ANOVA followed by Bonferroni *post-hoc* tests, mean ± SEM, *n* = 6–7.

The trafficking, insertion and stabilization in the recycling process are also critical components of normal AMPAR functions. To directly assess possible impact of LCX001 on altering the AMPAR recycling process and make real-time measurements of AMPA receptors on the surface of neurons, super ecliptic pHluorin (SEP) is used to visualize surface-expressed AMPA receptors containing GluA1 and GluA2(R) subunits on individual neurons. GluA2 or GluA1 tagged with super ecliptic pHluorin [SEP-GluA2(R) or SEP-GluA1] is expressed in cultured primary medulla oblongata neurons, and live cell imaging is used to record the change in absolute fluorescence in real time. When neurons, transfected with recombinant SEP-AMPAR adenovirus were prepared in the bathing solution containing artificial cerebrospinal fluid (ACSF, pH 7.4), surface stabilized SEP-GluA2 or SEP-GluA1 become relatively brightly fluorescent. In contrast, a sharp increase in fluorescence was caused by exposure to the bathing solution at a higher extracellular pH (8.5). Furthermore, the fluorescence intensity of SEP-GluA2 or SEP-GluA1 could be eclipsed after exposure to a rapidly acidified environment (pH 6.0). These data revealed that AMPARs containing SEP-GluA2 or SEP-GluA1 were successfully expressed in neurons, and that the fluorescence was sensitive to extracellular pH changes (Figure 5A). In the following tests, pHluorin fluorescence was quenched inside acidic vesicles, and they became fluorescent when the vesicles were exposed to the extracellular neutral or alkali solutions. The recycling and stabilization process could be detected as transient and dynamic changes in fluorescence at a basic extracellular pH (7.4). As a control group, application of vehicle (20% HPCD) had no significant impact on the fluorescence intensity of SEP-GluA2 or SEP-GluA1, and did not alter extracellular pH because of the buffering system in the bathing solution (Figure 5B,C). Similarly, CX614 treatment with a final dose of 47 μM resulted in no significant difference in SEP-GluA1 and SEP-GluA2 fluorescence compared with the beginning of the test (Figure 5D,E, *P* > 0.05). The presence of 47 μM LCX001 also exerted no impact on the fluorescence intensity of SEP-GluA1 (Figure 5D,E, *P* > 0.05). However, a significant and fast increase in SEP-GluA2 fluorescence was exhibited in response to application of LCX001 (final dose of 47 μM) for 6 min (Figure 5F,^∗^*P* < 0.05). The fluorescence intensity data and the relative intensity to vehicle group showed that application of LCX001 caused significant enhancement of stabilization and accumulation of AMPARs containing GluA2 units on the surface of neurons (Figure 5D,F,^∗^*P* < 0.05).

### LCX001 Regulates the Intracellular Calcium Concentration ([Ca^2+^]_i_) of Cultured Medullary Neurons in Response to Opioid Drugs

**FIGURE 6:**
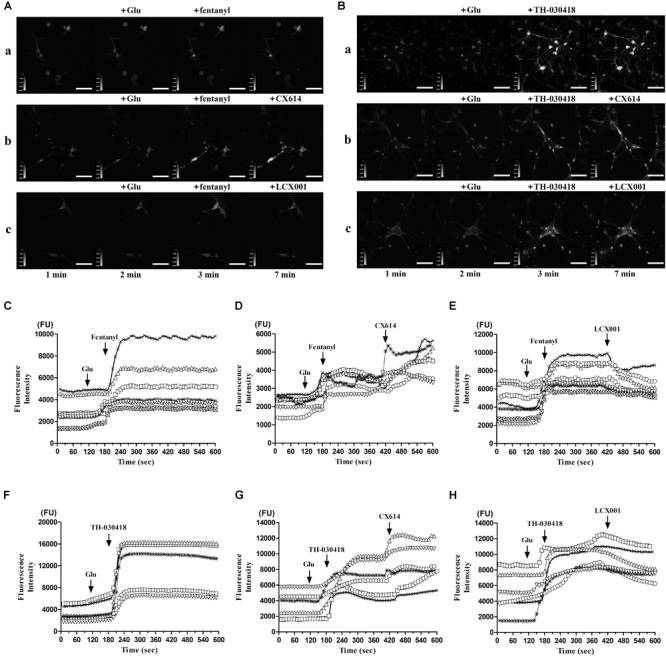
Variation of intracellular calcium concentration ([Ca^2+^]_i_) in response to opioid drugs and following application of LCX001 or CX614. Each trace in this figure was representative to continuous recording of a specific neuron by live cell imaging for 10 min. **(A,B)** Representative time courses of intracellular calcium fluorescence intensity were measured in Fluo-4 NW dye buffer-loaded primary cultured medulla oblongata neurons, *Scale bar* = 70 μm. The neurons were treated with glutamate, fentanyl/TH-030418 and LCX001/CX614 consecutively. Final compound concentrations and time points of application: glutamate (18 μM, 2 min), fentanyl or TH-030418 (42 μM, 3 min), then LCX001 or CX614 (40 μM, 7 min). **(C–E)** fentanyl caused a rapid rise in fluorescence intensity and [Ca^2+^]_i,_ which was blocked by LCX001 treatment and enhanced by CX614. **(F–H)** TH-030418 triggered an increase in fluorescence intensity and [Ca^2+^]_i._ The typical [Ca^2+^]_i_ rise was prolonged in response to CX614. In contrast, application of LCX001 was instrumental in suppressing the high level of [Ca^2+^]_i_. The time-lapse imaging was set at 1 s intervals for 10 min, *n* = 6–7.

The previous studies demonstrate that GluA2(R) subunit can undergo special Q/R RNA editing, which effectively reduces calcium influx. The majority of GluA2 subunits ( > 95%) contain the arginine (R) residue, and the restriction of intracellular calcium concentration by GluA2(R) is deemed vital to normal neuronal function. LCX001 may mediate [Ca^2+^]_i_ and neuron function by means of increasing surface GluA2(R) expression. Real-time detection of [Ca^2+^]_i_ in Fluo-4 NW dye buffer-loaded primary cultured medullary neurons is conducted in the presence of glutamate following application of opioid drugs and AMPA modulators, and the variation of fluorescence intensity is considered to represent changes of intracellular calcium concentration ([Ca^2+^]_i_). As shown in Figure 6A,B, in the control group, application of glutamate at a final dose of 18 μM stimulated a weak increase in [Ca^2+^]_i_. At the time point of 3 min, the fluorescence intensity was significantly enhanced after treatment with fentanyl or TH-030418 (final dose of 42 μM). This indicated that both fentanyl and TH-030418 triggered a rapid rise in [Ca^2+^]_i_ in nearly all parts of the neuron soma (Figure 6C,F). The remarkable [Ca^2+^]_i_ bursts lasted for 7 min and did not recover back to resting levels (Figure 6C,F). Interestingly, the increased level of [Ca^2+^]_i_ in response to fentanyl and TH-030418 treatment was significantly blocked by the subsequent application of LCX001 (final dose of 40 μM) at the time point of 7 min (Figure 6A,B,E,H). Compared to the reducing effect of LCX001 on [Ca^2+^]_i_, CX614 (final dose of 40 μM) continuously increased level of [Ca^2+^]_i_ (Figure 6A,B,D,G). These results revealed that LCX001 restrained calcium influx and mediated intracellular calcium concentration in response to opioid treatment.

## Discussion

Respiratory depression sometimes hinders the use of analgesics and sedative hypnotics. Here, among the RespireRx (Cortex) patents, we identify an ampakine compound, LCX001, that can alleviate suppressed respiration. LCX001 has a typical positive modulatory impact on AMPAR-mediated function. Importantly, LCX001 application generates a profound increase in GluA2(R) surface expression, and potently restrains the abnormal intracellular Ca^2+^ load, which is possibly a new action of ampakines in modulating breathing.

Typical analgesic and sedative compounds, including opioids, pentobarbital, and propofol, were selected to give rise to respiratory depression in rodents. With regard to varying degrees of respiration suppression by different targets, LCX001 (i.v.) at 10 mg/kg was simultaneously effective in rescuing and preventing depression. Furthermore, compared to effect against depression by pentobarbital, LCX001 restored rhythmic activity from severe inhibition by opioid drugs rapidly and exhibited a more pronounced impact. It was indicated that the variance existed in the therapeutic effect of LCX001 against different analgesics and sedative hypnotics induced respiratory depression at the same dose.

We next verified the pharmacological characteristics of novel AMPA receptor potentiator LCX001. From the saturation binding curves and whole cell patch-clamp, LCX001 facilitated the binding ability of both the exogenous agonist [^3^H]AMPA and the endogenous neurotransmitter glutamate in native membranes and on recombinant AMPA receptors, which was consistent with the properties of classical AMPA modulators (Arai et al., 2000; Lindén et al., 2001). Subsequent attempts were made to examine the potency of accentuated AMPAR-mediated conductance by recombinant GluA2-AMPARs expressed in HEK 293 Cells. A detectable increase in the peak amplitudes evoked by glutamate was readily and positively modulated with application of LCX001. Therefore, it is possible that LCX001 promoted the inward currents on the neurons controlling respiratory function and participated in protection from respiratory depression. In addition, the effect of typical AMPA modulator CX614 on glutamate-induced amplitude was also tested. Application of 100 μM CX614 increased the peak response of glutamate at both 3.5 and 10 mM, which is in accorded with previous studies. Compared to the properties of LCX001, CX614 even produced a higher modulatory impact on glutamate (3.5 mM)-induced stimulation (Figure 4E, ^∗^*P* < 0.05). Previous studies indicate that ampakines come in two broad classes with high and low impact. The former, like CX614 or CX546, are quite potent. However, there are concerns with the safety threshold for causing over-excitability of the central nervous system (CNS), and high impact AMPA modulators often obtain a relatively narrow therapeutic window (Lynch, 2006; Wang et al., 2012). Compared to high impact compounds, low impact ampakines are less likely to bring about severe safety hazards (Le et al., 2014). LCX001 performed a treatment effect on respiratory depression, and no evidence of over-excitability were observed in rodents after LCX001 administration (up to 60 mg/kg). LCX001 primarily increased glutamate-induced amplitude in the whole-cell recordings. From the results above, LCX001 might fit with the scheme of low impact ampakine compounds. However, more experiments should be proceeded to confirm precise property of LCX001 from its binding site of the AMPA receptor.

The unremitting recycling process of AMPARs plays a major role in their biological function (Wang et al., 2012). The trafficking, insertion and stabilization in this process significantly modulates the pharmacological properties of AMPA receptors (Turrigiano and Nelson, 2004; Marder and Goaillard, 2006; Hou et al., 2008). It is not clear whether AMPA modulator exerts impacts on the recycling process of AMPARs. The live cell imaging and super ecliptic pHluorin was used to dynamically analyze orientation and surface distribution of AMPARs in individual neurons in real-time (Kopec et al., 2006; Sanderson et al., 2011). By specifically imaging AMPA receptors at the neuron surface, application of LCX001 significantly enhanced accumulation of AMPARs containing SEP-GluA2(R) units but not SEP-GluA1 units. This finding indicated that LCX001 caused a rapid and steady increase in the number of surface GluA2 subunits and modulated the behavior of AMPA-GluA2 receptors. Compared to the unusual effect of LCX001, CX614 treatment caused no change in the fluorescence of SEP-GluA2(R) and GluA1, and did not alter AMPA receptor trafficking or surface stabilization. GluA2 subunits play a pivotal role in the biophysical properties of AMPA receptors. Predominant numbers of AMPARs in the central nervous system contain heteromers with GluA2(R), and special RNA editing at position 607 within the M2 membrane loop region commonly results in a positively charged arginine (R) residue replacing the uncharged glutamine (Q). This GluA2 Q/R editing is specific and impermeable to extracellular calcium influx (Sans et al., 2003; Isaac et al., 2007). We conjectured that the enhanced surface expression of GluA2(R) triggered by LCX001 might regulate intracellular calcium concentration. From the Ca^2+^ imaging of medullary neurons, a significant rapid rise of fluorescent intensity was produced after fentanyl or TH-030418 application and the rise was stable for at least 7 min. These data indicated that fentanyl or TH-030418 could prominently enhance and up-regulate intracellular [Ca]_i_. Opioids could activated phospholipase C (PLC) and inositol 1,4,5-triphosphate (IP_3_) to increase cytoplasmic calcium, which were consistent with those of previous studies (Wandless et al., 1996; Spencer et al., 1997; Wang et al., 2000). We suggested that the elevated intracellular calcium participated in the formation of opioid-induced respiratory depression. L-type Ca^2+^ channels were found in the in medullary neurons of mammals, and respiratory function was directly mediated by this Ca^2+^ channel to a certain extent (Mironov and Richter, 1998). An agonist of the L-type of Ca^2+^ channel in the ventral medullary area of the cat could trigger dose-dependent respiratory depression (Ruiz et al., 1993). The L-type Ca^2+^ channel agonist directly facilitated channel opening, which effectively promoted calcium influx (Bellemann and Franckowiak, 1985; Middlemiss and Spedding, 1985), and typical respiratory depression was prevented by treatment with the channel antagonist, nimodipine (Ruiz et al., 1993). These results indicated that increased cytoplasmic calcium might be one of the causes to trigger respiratory depression. The elevation of intracellular calcium caused by opioids might contribute to depression in the medullary neuron. Different from the effect of opioids, LCX001 selectively recruited Ca^2+^-impermeable GluA2(R) subunits and restrained the possible calcium influx activated by glutamate receptors. The high levels of intracellular calcium concentration remained unchanged or partly declined after LCX001 application. This unique effect might stabilize calcium mobilization and impart neuroprotection. Compared to the special effect of LCX001, when the neurons had high levels of calcium, CX614 application still triggered a higher level of calcium inflow on account of nonselective and positive modulation on Ca^2+^-permeable AMPA subunits (GluA1/3/4) in the presence of glutamate. Ca^2+^ homeostasis was necessary for neuronal communication and survival. This abnormal intracellular Ca^2+^ load might bring about excitotoxic lesions, which partly accounted for potential safety hazard of hyper-excitability of high impact ampakine CX614.

AMPA receptor signaling has an impact on pro-nociceptive or anti-nociceptive formation (Gear et al., 1999; Ghalandari-Shamami et al., 2011). The previous data demonstrated that CX546 prominently alleviated pain hypersensitivity and depression-like behavior in the spared nerve injury (SNI) model of neuropathic pain as well as complete freund’s adjuvant (CFA) model of inflammatory pain of rats (Le et al., 2014). In addition, administration of CX546 into nucleus accumbens (NAc) provided antinociceptive effect in paw incision (PI) model of acute pain and inhibited persistent postoperative pain from the SNI model (Su et al., 2016). When injected locally into the prefrontal cortex (PFC), CX546 also reveal synergistic effect to morphine analgesia (Sun et al., 2017). The results indicated that analgesia effect of CX546 might be involved in the acting through AMPA receptors in the NAc and PFC. Only a few ampakine compounds has been reported to express potential analgesic properties. Our data revealed that LCX001 also performed prominent anti-nociceptive effects in systemic administration (intravenously) in rodents, and revealed certain analgesic actions altering the threshold of acute pain. Compared to morphine, the favorable analgesic properties of LCX001 might make it applicable for pain relief. From the results of live cell imaging, the upregulation of GluA2(R) by LCX001 treatment on the neuron surface might potentiate the anti-nociceptive effect. Previous studies indicated that postoperative and inflammatory pain markedly enhanced GluA1 AMPA receptor subunits on the membrane, whereas the delivery of GluA2 subunits could be inhibited and decreased (Katano et al., 2008; Park et al., 2009). The accelerated GluA2-lacking AMPA receptor formation generated and amplified pain transduction (Hartmann et al., 2004; Guo et al., 2014). Thus, the promotion of surface GluA2(R) to restrict pain transmission might be a mechanism for analgesia efficacy of ampakine LCX001.

## Conclusion

In conclusion, the ampakine LCX001 provides protection against suppression of respiration from analgesics and sedative hypnotics, and also performs significant anti-nociceptive effects in rodent models. The typical positive regulating effect and potential new pharmacological properties of LCX001 may be beneficial for pain therapy and has the potential to be used in conjunction with future treatment approaches to alleviate respiratory depression.

## Author Contributions

WD, XG, and ZY carried out the experiments. ZY and R-BS conceived the project and supervised the research. DX and X-BZ executed the chemical synthesis. Y-LL accomplished the construction of stable cell line. WD and ZY wrote the manuscript. All authors discussed the results and commented on the manuscript.

## Conflict of Interest Statement

The authors declare that the research was conducted in the absence of any commercial or financial relationships that could be construed as a potential conflict of interest.
